# Case of a Paralytic Affection, Occasioned by the Calx of Lead, and Cured by Electricity

**Published:** 1784

**Authors:** 


IV. *Caſe of a paralytic affection, occaſioned by the calx of lead, and cured by electricity. Communicated to Dr.* Simmons, F. R. S. *by Mr.* John Whitehurſt, F. R. S.

THE facts relative to the removal of a paralytic affection occaſioned by the calx of lead, and of which you requeſted me to give you an account, are as follows:

Thomas

Thomas Yopp, a labouring man, now living at Derby, was employed in a red lead work near that place, in which fervice he was accuftomed to perform the operation of feparating the calcined parts of the lead from thofe not completely fo, by ftirring the lead about in a tub of water with his hand; by which means the part became totally deprived of fenfation and mufcular motion, and remained in this ftate for the fpace of two years. At the end of that time electrical fhocks were gently applied from the wrift to the ends of the fingers, but without the leaft degree of fenfation being produced in the part. The fhocks were therefore increafed gradually till the contents of a large Leyden bottle, as highly charged as poffible, were paffed through the hand. The effects produced by this laft operation were a warmth in the part, a profufe fweat, and a tingling in the fingers. The hand was then wrapped in flannel, and the man ordered to return the next day to have the operation repeated; but he returned no more, having acquired fo much ftrength in his hand as enabled him the following day to take a burthen from his head, of near feventy pounds weight.

The above operation was performed in the year 1769, and the part continues perfectly well, well, the little finger excepted; and I have ſome reaſon to believe that the electrical fluid did not paſs through it.

Though the man acquired ſo much ſtrength the firſt night after the ſhocks were adminiſtered; yet two or three months elapſed before his hand recovered its uſual ſtrength.

London, March 12, 1784.

